# Bedside ultrasound in cardiac standstill: a clinical review

**DOI:** 10.1186/s13089-019-0150-7

**Published:** 2019-12-30

**Authors:** Laila Hussein, Mohammad Anzal Rehman, Ruhina Sajid, Firas Annajjar, Tarik Al-Janabi

**Affiliations:** 1 Mafraq Hospital, Abu Dhabi, United Arab Emirates; 2 Zayed Military Hospital, Abu Dhabi, United Arab Emirates; 3 Mediclinic Hospital, Dubai, United Arab Emirates; 4 Rashid Hospital, Dubai, United Arab Emirates

**Keywords:** Ultrasound, Cardiac arrest, Resuscitation, Cardiac standstill, Point-of-care ultrasound, Echocardiography, Transesophageal echocardiography, Predictors of survival in cardiac arrest

## Abstract

Patients with cardiac arrest present as a relatively frequent occurrence in the Emergency Department. Despite the advances in our understanding of the pathophysiology of cardiac arrest, managing the condition remains a stressful endeavor and currently implemented interventions, while beneficial, are still associated with a disappointingly low survivability. The majority of modern Advanced Life Support algorithms employ a standardized approach to best resuscitate the ‘crashed’ patient. However, management during resuscitation often encourages a ‘one-size-fits-all’ policy for most patients, with lesser attention drawn towards causality of the disease and factors that could alter resuscitative care. Life support providers are also often challenged by the limited bedside predictors of survival to guide the course and duration of resuscitation. Over the recent decades, point-of-care ultrasonography (PoCUS) has been gradually proving itself as a useful adjunct that could potentially bridge the gap in the recognition and evaluation of precipitants and end-points in resuscitation, thereby facilitating an improved approach to resuscitation of the arrested patient. Point-of-care ultrasound applications in the critical care field have tremendously evolved over the past four decades. Today, bedside ultrasound is a fundamental tool that is quick, safe, inexpensive and reproducible. Not only can it provide the physician with critical information on reversible causes of arrest, but it can also be used to predict survival. Of note is its utility in predicting worse survival outcomes in patients with cardiac standstill, i.e., no cardiac activity witnessed with ultrasound. Unfortunately, despite the increasing evidence surrounding ultrasound use in arrest, bedside ultrasound is still largely underutilized during the resuscitation process. This article reviews the current literature on cardiac standstill and the application of bedside ultrasound in cardiac arrests.

## Background

Cardiac arrest is one of the leading causes of death and disability worldwide. Unsurprisingly, patients in arrest are frequently encountered in the Emergency Department (ED) and life-saving resuscitation techniques constitute an integral part of every Emergency Healthcare provider’s core training. Cardiopulmonary resuscitation in the ED is an intense event that paralyzes the resuscitation room and requires expeditious decision-making and rapid intervention. Although medical personnel are accustomed to the swift execution of assigned tasks in the initial few minutes, the majority of time during resuscitation is spent either repeating initial actions or waiting for the next development which, unfortunately, often tends to be the announcement of time of death. According to the American Heart Association 2013 statistics, overall survival to hospital discharge rate was 9.5% and 23.9% for out-of-hospital arrest and in-hospital arrest, respectively [1]. Out-of-hospital cardiac asystole often carries the worst prognosis with a survival rate of only 2–7% [2].

Despite the lack of evidence to support such practice, many Emergency Physicians and hospital policies have adopted and implemented 20 min as the average time spent on cardiopulmonary resuscitation (CPR) before termination [3]. While it is worrying that allotting a standard time across all patients in arrest might leave some patients with sub-optimal care during arrest, what is more concerning is that this premature termination of CPR is based on limited bedside decision tools. While it is true that, on average, resuscitation is often unnecessarily prolonged, consumes a great deal of hospital resources and has contradicting impact on family members [4], certain populations of patients may warrant and ultimately benefit from longer resuscitation efforts such as in cases of pediatric arrest, drowning, and hypothermia.

Several factors associated with prognosis in patients undergoing cardiac arrest have also been studied. Better prognosis has been reported in patients with initial shockable rhythm, bystander CPR, witnessed arrest, and in patients who are conscious on admission [5, 6]. Patients with agonal movements and gasping during resuscitation and those with end tidal carbon dioxide (ETCO_2_) values higher than 10 mmHg were also found to have better outcomes [7, 8]. Unfortunately, up to date, there are no 100% accurate predictors that can definitively determine which patients will have return of spontaneous circulation (ROSC) and which will not.

For the past three decades, literature has revealed that point-of-care ultrasound (PoCUS) in cardiac arrest may add a significant prognostic value to the currently available clinical exam, specifically pertaining to ROSC. The use of imaging during CPR dates back to the 1980s when transesophageal echocardiography was first used to look for reversible causes of arrest [9, 10]. Since then, the application of ultrasound in cardiac arrest has widely expanded and become a core skill recognized by many international organizations [11, 12]. One of the applications of ultrasound in cardiac arrest involves identifying the complete absence of cardiac motion, termed cardiac standstill. Current literature has shown very low (but not zero) chance of survival associated with a cardiac standstill [2].

## International consensus on the use of POCUS in CPR

In 2010, the American Society of Echocardiography and the American College of Emergency Physicians established a consensus on cardiac ultrasound applications in the emergency department. The goal of focused ultrasound in cardiac arrest is to (1) differentiate organized cardiac rhythm from asystole, true PEA (pulseless electrical activity) and pseudo-PEA; (2) find reversible causes of arrest and (3) perform ultrasound-guided procedures during CPR and in ROSC [11]. The European Society of Cardiology stated that ultrasound may potentially improve diagnosis and alter management throughout the whole pathway of acute care in cardiac arrest patients [12]. Strong recommendations were also empowered by the European Resuscitation 2015 Guidelines and incorporated ultrasound into Acute Life Support [13]. Finally, in 2017, ACEP approved guidelines on transesophageal echocardiography (TEE) use in cardiac arrest [14].

## Level of competency

Use of ultrasound during CPR requires a certain degree of experience and training. Operators should be practiced in obtaining different heart windows in stable patients first and need to be capable of correctly interpreting different pathologies. They should have considerable training integrating the scan into advanced life support without delaying chest compressions. No specific hours of training were postulated in the European Heart Society and European Resuscitation Council Guidelines [12, 13]. However, several studies have demonstrated accurate cardiac ultrasound skills by emergency medicine physicians as well as emergency medicine residents with good correlation to cardiologists’ skills [15–17]. One study found that the implementation of a standardized 6-month course for emergency healthcare providers allowed them to utilize bedside ultrasonography to efficiently obtain useful prognostic indicators of survival and ROSC while still maintaining between-compression delays of less than 10 s [18].

## Applied cardiac arrest ultrasound

### Ultrasound should not delay chest compressions

When a patient arrives to the Emergency Department in cardiac arrest, looking for reversible causes should only be initiated after high-quality CPR has been achieved and maintained. That being said, PoCUS should not be delayed till the very end of resuscitation as patients have higher chances of survival if potentially reversible causes of arrest were identified and addressed early. Although ultrasound use in arrest is strongly encouraged, users need to be aware of potential harm if not applied correctly. Most importantly, ultrasound application in cardiac arrest should never interrupt or interfere with chest compressions [12, 13].

In 2017 and 2018, three studies demonstrated that use of ultrasound increases hands-off time during pulse checks [19–21]. Among these was a prospective cohort study by Huis and his colleagues where cameras were installed in the resuscitation room as a means of visually monitoring any and all interruptions during CPR [19]. Twenty-three patients were enrolled in the study with a total of 123 pulse checks. The mean duration of pulse checks and interruption in chest compressions with PoCUS was found to be 21.0 s (95% CI 18–24), compared with a mean duration without PoCUS of 13.0 s (95% CI 12–15). Thus, the study demonstrated that the use of PoCUS significantly increased the pulse-check duration by 8.4 s (95% CI 6.7–10.0 [p < 0.0001]). Similar findings were recorded by Clattenburg et al. [21]. It is important to emphasize that none of these studies advised against the use of ultrasound but rather advocated for sensible techniques and proper training.

### Image acquisition

The first algorithm incorporating cardiac ultrasound into CPR was introduced by Breitkreutz et al. and was described as focused echocardiographic evaluation in resuscitation (FEER) [22]. Breitkreutz recommends FEER after 5 cycles of high-quality CPR. Integrating POCUS into cardiac arrest protocols was also suggested by several other authors over the years [23–25]. We recommend using ultrasound earlier in CPR once high-quality chest compressions are achieved and then repeating it prior to termination of CPR to assess if the patient is having a cardiac standstill. Ultrasound also plays a key role after ROSC has been achieved. Reversible causes of shock that are otherwise usually unclear during CPR can be readily identified through the use of ultrasound—such as wall motion abnormalities in patients with acute myocardial infarction. Ultrasound can also guide fluid management during ROSC by scanning the inferior vena cava and reassessing the lungs for development of pulmonary edema.

**Fig. 1:**
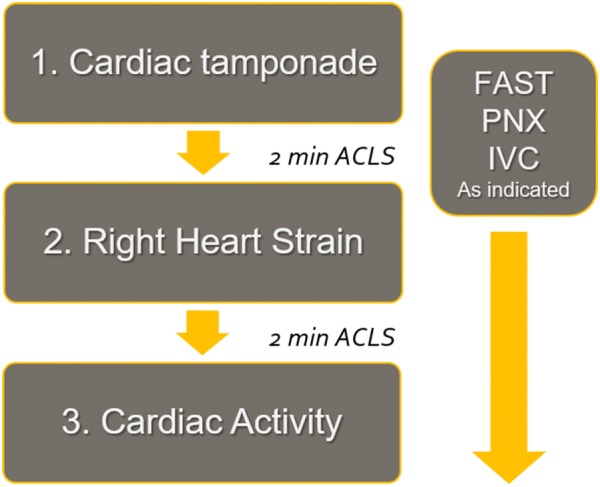
The Cardiac Arrest Sonographic Assessment (CASA) protocol. Reproduced with author’s permission from the original algorithm curtesy of Gardner et al. [26]

In 2018, Gardner and Clattenberg et al. published a novel protocol known as Cardiac Arrest Sonographic Assessment (CASA) [26]. The authors postulated a three-step ultrasound-guided assessment of patients in cardiac arrest. Initial image acquisition focuses on ruling out cardiac tamponade. The second image attempts to rule out right ventricular strain secondary to pulmonary embolism. The third view is the final step of the protocol, performed at the end of resuscitation to identify cardiac standstill (Fig. 1). Other windows commonly included in the EFAST exam can be performed while the CPR is ongoing. The CASA protocol was tested through a prospective pre- and post-intervention study on 276 cardiac arrests where the emergency medicine residents and faculty had been trained to apply the protocol during CPR [27]. The CASA group had significant reduction in pulse check interruptions from 19.8 to 15.8 s. The study also showed that placing the ultrasound on the chest prior to stopping CPR and having an attending with ED ultrasound fellowship training are independent variables that reliably decrease CPR pulse check durations. Although further external validation of this protocol is warranted, its simplicity renders it an attractive tool to integrate into the resuscitation process.

**Fig. 2:**
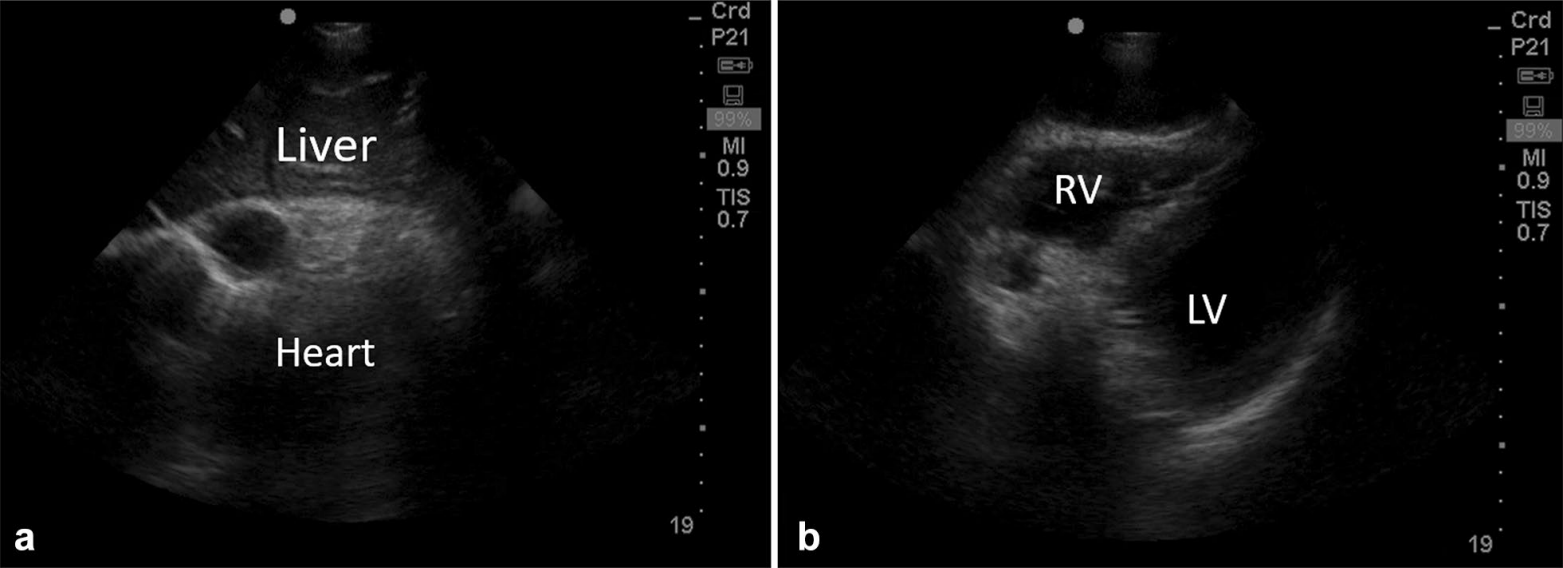
Subcostal views of the heart taken while chest compressions were ongoing. **a** The heart was compressed (systole) and chambers are difficult identify. **b** The hands are off the chest and the right (RV) and left (LV) ventricles are seen clearly

**Fig. 3:**
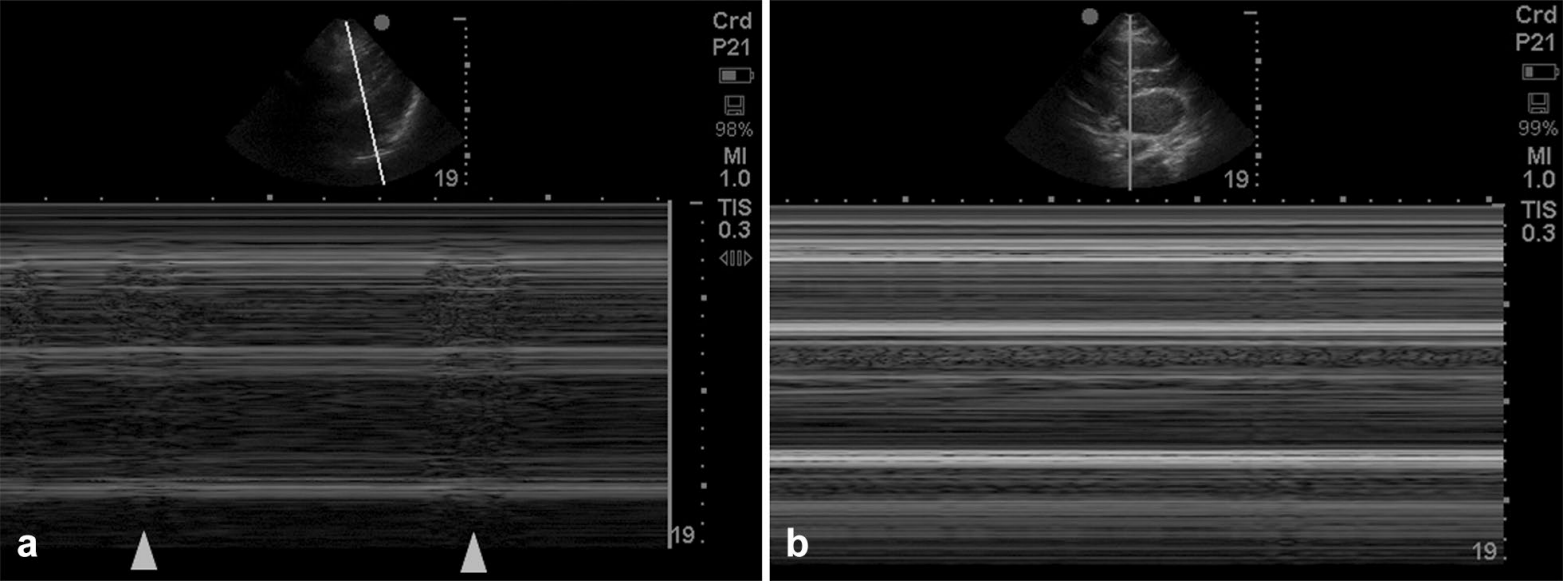
M-mode of the heart in asystole. **a** The horizontal lines represent absence of any movement across the M-mode line. Areas which are grainy (arrows) indicate myocardial contraction. **b** Completely flat lines indicating no cardiac movement (true cardiac standstill)

In order to optimize image acquisition, the heart window may be obtained while CPR is still ongoing prior to the pulse check (Fig. 2a, b) [27]. The operator can then record the heart activity during the pulse check and review it when CPR has resumed. This can be repeated on the next pulse check if an adequate image was not obtained the first time. If no heart activity is seen, M-mode can be used to confirm the presence or absence of any cardiac movement (Fig. 3a, b). Only one heart window should be attempted at a time. It is also recommended that a designated team member be assigned to verbalize a loud ‘count-down’ during the scan to minimize between-compression delays. The operator performing the scans can also keep towels and tissues ready to immediately remove the gel off the chest before resumption of compressions. Table 1 summarizes the key points to faster image acquisition.

**Table 1:** Key points in faster image acquisition during CPR

Minimizing compression interruptions
Establish high-quality CPR
Presence of at least 2 physicians in the code
The most experienced user should scan
Get ready with a towel in your hand
Choose your probe and presets ahead
Acquire images before the pulse check
Have someone do a 10-s count down
Only 1 window per pulse check
Record your scan and review later

**Fig. 4:**
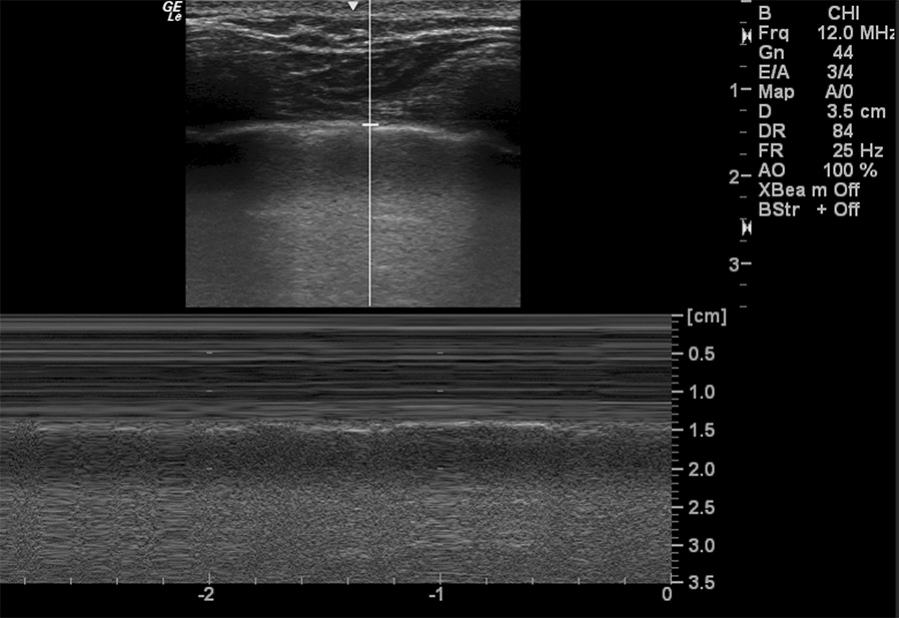
Parasternal view of the lungs in M-mode showing sea shore appearance that confirms absence of pneumothorax

The phased array (cardiac) probe is ideal for scanning the heart. However, if not available, the curvilinear (abdominal) probe can also be used. Three cardiac windows, subcostal, apical, and parasternal long axis views, have been described in cardiac arrest literature, but any one view is usually sufficient if it provides the operator with all the answers he is looking for [12, 28]. The subcostal view is most popular as it can be most easily accessed during ongoing CPR without interfering with chest compressions [24]. In addition to cardiac windows, a quick view of the lungs can be obtained during another pulse check to look for absent lung sliding (Fig. 4).

## Scanning objectives

### Identification of reversible causes

Hernandez et al. developed a systematic algorithm to identify the four most critical causes of cardiac arrest in the C.A.U.S.E. protocol (Cardiac Arrest Ultra-Sound Exam) [24]. These potentially reversible causes include a dilated right ventricle in pulmonary embolism, fluid around the heart in pericardial tamponade, collapsed ventricles in hypovolemia and absent lung sliding in tension pneumothorax. The authors have also suggested additional views that can be obtained in hypovolemia such as the inferior vena cava to confirm an ‘empty tank’, as well as the abdominal aorta to evaluate for aneurysm as a cause of hypovolemia.

The American Society of Echocardiography and the American College of Emergency Physicians 2010 guidelines recommend the use of PoCUS only in pulseless electrical activity (PEA) or asystole and discourage its use in shockable rhythm [11]. Their justification is rational as identification of ventricular fibrillation or pulseless ventricular tachycardia should be followed by immediate shock delivery and resumption of chest compressions. Detecting pathologies such as wall motion abnormality or hypertrophic cardiomyopathy is unlikely to affect the management during CPR, but should be identified after ROSC. However, we believe there are exceptions where ultrasound might be valuable in such scenarios, especially if the ventricular fibrillation is refractory. Cardiac arrest due to pulmonary embolism can present with ventricular fibrillation in 5% of cases [29]. Ultrasound in this scenario may prompt the physician to administer thrombolytic therapy. Patients with wall motion abnormality in refractory ventricular fibrillation might benefit from coronary intervention even with ongoing CPR [29].

### Right ventricular heart strain in cardiac arrest

A number of studies incorporating PoCUS in cardiac arrest mention investigation of the right ventricle for dilatation to evaluate for the presence of a pulmonary embolism [11, 24]. However, an increasing amount of evidence suggests that this may be an ineffective method to rule in the diagnosis. While it has been well established that the presence of a pulmonary embolus can undoubtedly result in right heart strain that could manifest as a dilated RV, a number of other factors, such as hypovolemia, hyperkalemia, primary arrhythmias and pre-existing chronic right ventricular strain, have also been shown to produce similar right-sided enlargement during cardiac arrest [30–32].

In fact, an interesting aspect to consider is the phenomenon that some degree of RV dilatation may even be a normal consequence of resuscitation during arrest. Gabriel Wardi et al. found that RV strain and dilatation was more demonstrable when a greater amount of time had elapsed into resuscitation [33].

The 2019 European Society of Cardiology guidelines on pulmonary embolism have addressed the accuracy of RV dilatation in pulmonary embolism [34]. Although RV dilatation plays an important prognostic role in stable patients with pulmonary embolism, it has a rather weak positive predictive value for PE-related deaths. This frequently encountered relative insensitivity is partially attributable to the inherent difficulty in standardizing ultrasound parameters for any study [34].

In light of the presence of such false-positives when visualizing the right side of the heart during cardiac arrest, the diagnosis of pulmonary embolism and any subsequent intervention based on the same should be further augmented by factors other than isolated right heart strain on PoCUS. Historical details of pre-arrest signs and symptoms, as well as possible intra-arrest evaluations for deep vein thrombosis in high-risk patients could prove useful measures to dependably diagnose and treat pulmonary embolism during cardiac arrest [35]. Furthermore, increased presence of false-positive findings late into resuscitation prompts consideration of integrating ultrasound evaluations as early as possible into cardiac arrest protocols.

### Identification of cardiac standstill

Cardiac standstill, also known as *true asystole*, is defined as the complete absence of any cardiac motion including the ventricles, atria and valves [36, 37]. Patients recognized to have standstill with concomitant electrical activity on the monitor are often described to have *true PEA*. *Pseudo*-*PEA* is the presence of ventricular contractility visualized by ultrasound with electrical activity but no palpable pulse [11]. The M-mode option on ultrasound detects any motion along a given line against time. If any movement is identified, that part of the heart will look hazy like “sand on a beach” (Fig. 3a). When there is a complete absence of cardiac contractility, the image will resemble a “barcode” appearance (Fig. 3b).

Identifying true PEA or cardiac standstill on ultrasound carries an important prognostic value. In 2001, Blaivas et al. conducted one of the earliest and largest prospective studies on cardiac standstill [38]. Of the 169 patients, 136 were found to be in standstill and had 0% survival regardless of the electrical rhythm they presented with. On the other hand, 20 patients survived to hospital admission out of 33 patients with cardiac activity on initial ultrasound. Mean patient age in this study was 71 years which may admittedly represent a more senior population than is usually encountered in other centers. The study’s considerable limitation was that it included only out-of-hospital arrests where overall survival is less than with in-hospital arrests. No data was provided about survival to hospital discharge or neurological outcomes.

Salen et al. had similar outcomes in his two prospective studies in 1999 and 2005 [36, 37]. However, in the earlier study, out of 59 patients with no cardiac activity, 2 had survived. Several other studies have been published during the last two decades with similar findings of poor outcome associated with cardiac standstill, but most of them still reported ROSC incidence in a small number of patients with cardiac standstill [39–42]. One of the highest survivals of patients with no wall motion was reported by Breitkreutz et al. in 2010 wherein a total of five (10%) out of 50 patients with no cardiac movement survived [28]. His results also confirmed that the presence of wall motion can predict a much higher survival rate (*n* = 30/75, 40%). This was further validated in more recent study, the US-CAB protocol where cardiac activity identified in 47 cases (26.6%) out of a total of 177 arrest patients being studied was associated with higher rates of ROSC (95.7% vs. 21.5%, *p* < 0.0001) and survival to hospital discharge (25.5% vs. 10.0%, *p* < 0.01). Furthermore, detection of cardiac activity after 10 min of CPR exhibited 100% sensitivity, specificity, positive and negative predictive value for ROSC [18].

The largest, multi-center, observational prospective study was published in December 2016 by Gaspari et al. [2]. The REASON 1 trial (Real-time Assessment and Evaluation with Sonography—Outcomes Network), included over 20 hospitals and enrolled 793 patients. The study looked at rate of ROSC, rate of survival to hospital admission and to hospital discharge. Overall survival to discharge was 0.6% (*n* = 5) for patients in cardiac standstill and 3.8% for patients with cardiac activity (*n* = 30). A subgroup analysis of patients who had no bystander CPR, presented with asystole and had no cardiac activity on arrival, had no ROSC. Furthermore, REASON was the first trial to prove that sonographic identification and treatment of a reversible cause of a cardiac arrest increases survival. Fifteen percent of identified pericardial tamponades in the trial achieved ROSC and were discharged out of the hospital.

In March 2019, Members of the Sonography in Hypotension and Cardiac Arrest (SHoC) Investigators published a meta-analysis on the reliability of PoCUS to predict outcome in non-traumatic out-of-hospital and in-hospital cardiac arrests [43]. Ten studies with 1486 participants were included. Presence of cardiac activity on PoCUS had a pooled sensitivity of 60.3% (95% CI 38.1–78.9%) and specificity of 91.5% (80.8–96.5%) for ROSC. In asystole, the sensitivity of cardiac activity on PoCUS for predicting ROSC was 26.1 (7.8–59.6%) compared with 76.7% (61.3–87.2%) in PEA. Cardiac activity on PoCUS had higher odd ratios of 16.9 for ROSC, 10.3 for hospital admission and 8.03 for hospital discharge. Unlike previous metanalyses by Blyth et al. Tsou et al. and Wu et al. the SHoC group excluded studies with traumatic arrests or shockable rhythms [44–46]. One of the major discrepancies among the included studies was the definition of cardiac activity and operator experience.

Physicians who use bedside ultrasound in their resuscitations feel more comfortable terminating codes if no cardiac activity is found [47]. Although the chances of survival for cardiac activity in asystole are small, they are still three times higher than those in true standstill. Therefore, it would stand to reason that those particular patients would benefit more from longer resuscitation efforts.

## Future of cardiac arrest ultrasound

Point-of-care ultrasound is a science that is rapidly expanding with novel applications being published every year. POCUS may be applied to guide the quality of compressions by directly visualizing the contractility of left ventricle and adjusting hand placement [48, 49]. Ultrasound may also be used for pulse check between cycles of CPR. A few studies in the past have demonstrated that manual pulse check has poor sensitivity and specificity, with some studies showing accuracy of pulse check to be as low as 15% when limited to the 10 s permitted [50–52]. Ultrasound definitely offers much more reliable answers regarding cardiac output by direct visualization of ventricular contractility. Finally, there is a growing body of evidence supporting the use of TEE in cardiac arrest. TEE can provide the team with live feedback on cardiac status through the entirety of the resuscitation process. It offers better resolution of images, is applicable to all body habitus, and may limit breaks in chest compressions as image acquisition is much faster when compared to transthoracic cardiac ultrasound [51–55].

## Conclusion

Cardiopulmonary resuscitation has had a humble evolution over the last decade, but remains to have rather unfruitful results. For the most part, the physician involved has little information about the reason of arrest and likelihood of survival. This is where bedside ultrasound shows promise as a fundamental tool that can provide the physician with valuable information mandating change in management. Ultrasound can guide the resuscitative process under direct vision rather than by blind adherence to resuscitation protocols. An important point evidenced by most of the studies referenced above is the fact that cardiac motion visualized on ultrasonography is the best predictor of survival, and its use can provide much needed prognostic information that aids resuscitation. On the other hand, patients shown to have cardiac standstill on ultrasound have been shown to have very low chances of survival. Still, it is important to remember that the decision to terminate resuscitation should never be taken based on ultrasound findings alone. In summary, current CPR guidelines should not involve a ‘one-size-fits-all’ strategy for arrest patients. The decision to terminate needs to be tailored on a case-by-case basis. Point-of-care ultrasound can support this decision and needs to be adopted as part of the standard of care.

## Data Availability

Not applicable.

## Abbreviations

PoCUS: point-of-care ultrasonography
ED: Emergency Department
CPR: cardiopulmonary resuscitation
ETCO_2_: end tidal carbon dioxide
ROSC: return of spontaneous circulation
RV: right ventricle
PEA: pulseless electrical activity
TEE: transesophageal echocardiography
FEER: focused echocardiographic evaluation in resuscitation
CASA: Cardiac Arrest Sonographic Assessment
